# A portable image-based cytometer for rapid malaria detection and quantification

**DOI:** 10.1371/journal.pone.0179161

**Published:** 2017-06-08

**Authors:** Dahou Yang, Gowtham Subramanian, Jinming Duan, Shaobing Gao, Li Bai, Rajesh Chandramohanadas, Ye Ai

**Affiliations:** 1 Pillar of Engineering Product Development, Singapore University of Technology and Design, Singapore, Singapore; 2 School of Computer Science, University of Nottingham, Nottingham, United Kingdom; 3 School of Life Science and Technology, University of Electronic Science and Technology of China, Chengdu, China; Université Pierre et Marie Curie, FRANCE

## Abstract

Increasing resistance by malaria parasites to currently used antimalarials across the developing world warrants timely detection and classification so that appropriate drug combinations can be administered before clinical complications arise. However, this is often challenged by low levels of infection (referred to as parasitemia) and presence of predominantly young parasitic forms in the patients’ peripheral blood. Herein, we developed a simple, inexpensive and portable image-based cytometer that detects and numerically counts *Plasmodium falciparum* infected red blood cells (iRBCs) from Giemsa-stained smears derived from infected blood. Our cytometer is able to classify all parasitic subpopulations by quantifying the area occupied by the parasites within iRBCs, with high specificity, sensitivity and negligible false positives (~ 0.0025%). Moreover, we demonstrate the application of our image-based cytometer in testing anti-malarial efficacy against a commercial flow cytometer and demonstrate comparable results between the two methods. Collectively, these results highlight the possibility to use our image-based cytometer as a cheap, rapid and accurate alternative for antimalarial testing without compromising on efficiency and minimal processing time. With appropriate filters applied into the algorithm, to rule out leukocytes and reticulocytes, our cytometer may also be used for field diagnosis of malaria.

## Introduction

Malaria, one of the most devastating infectious diseases around the globe, is caused by protozoan parasites of the genus *Plasmodium*. There are five major species of plasmodia that infect humans, out of which *Plasmodium (P*.*) falciparum* causes the majority of morbidity and mortality in Africa followed by less lethal *P*. *vivax* infections across South-East Asia, altogether infecting 200 million people and resulting in over half a million deaths every year [1]. Currently used antimalarials include chloroquine, artemisinin sulfadoxine-pyrimethamine combination, atovaquone and clindamycin. However, parasites have acquired resistance to most of the above-mentioned drugs both in Africa and South-East Asia, rendering them inefficient for future usage [2–4].

Early diagnosis and treatment are required to avoid anemia, organ failure [5] and malaria-associated deaths [6]. Lack of reliable methods and tools in the field settings provide tremendous impedance to early diagnosis in malaria endemic areas such as sub-Saharan Africa. Traditional and widely practised method of malaria diagnosis relies on observable clinical symptoms associated, which are results of general host response to an infection. Undoubtedly, this method foists on several challenges due to the nonspecific nature of the symptoms that could be potentially caused due to other immune challenges, which could ultimately result in inappropriate and unnecessary exploitation of antimalarials [7, 8]. Rapid Diagnostic Tests (RDTs) used in clinics measure the presence of parasitic antigens such as aldolase or lactate dehydrogenase [9, 10]. However, these methods rely on detection of antigens derived from the parasites, rather than detecting the parasites themselves. Though RDTs are often able to differentiate most malarial species by means of their antigenic properties, overall sensitivity of detection is far below the threshold of microscopy-based malaria detection, exhibiting huge variations among the patients [11–15] and failing to guide treatments which could be fatal when mixed infections occur [16]. These are efficient methods; however the results can vary depending on the severity of infection and the high occurrence of young parasitic forms (ring-stage infections) in the peripheral blood, which are only beginning to establish metabolic processes. Due to the recent advancements in technology, several malaria diagnostic techniques such as microarray [17], PCR [18], loop-mediated isothermal amplification (LAMP) [19], flow cytometry [20], hemozoin detection using automated hematology analyser [21] have been developed for efficient malaria diagnosis. Diagnostic methods leveraging PCR, 18s-rRNA detection [22], mitochondrial cytochrome b activity [23, 24], *Pg*Mt19 and *Pf*MT869 mitochondrial regions [25], and the *Pvr47* and *Pfr364* genes [26, 27], have been used for detecting *Plasmodium* species. However, quantitative amplification of genes demands careful processing of blood to remove the inhibitors of amplification and thermal cycling, making it a cumbersome procedure. Notably, all of these methods demand expensive laboratory facilities and highly trained personnel to conduct complicated analytical procedures and data analyses, which may not be feasible in resource-poor malaria endemic countries.

The current gold standard technique for detecting malaria is microscopic examination of Giemsa-stained thin and thick blood smears [28] both in the field as well as in laboratory. This method allows detection of densely stained parasites against a background of lightly stained RBCs and widely accepted owing to cost effectiveness, simplicity and rapidity. Though, this method detects parasitemia levels of up to 1 infected cell in 10^6^ cells [29], the microscopic examination requiring traditional bulky microscope is laborious and often fails when the parasitemia is low, a situation very common in the case of *P*. *vivax* infections. In addition, logistic issues and challenges associated with transporting the traditional bulky microscopes to remote and rural malaria endemic regions [30] and fulfilling operational and maintenance requirements could be challenging. Furthermore, manual counting from Giemsa-smears is known to vary depending on the personnel engaged and quality of smears involving a high chance of misinterpreting other microorganisms such as bacteria and fungi as *Plasmodium* parasites and difficulties associated with identifying different *Plasmodium* strains [31]. Previously, several laboratories explored image processing based automated cell counting for parasitemia estimation, but most of them suffered from high false positive values [32–34], inferior accuracy [35], inability to differentiate parasitic stages [36] primarily rings [37] and the requirement of fluorescent dyes [38, 39].

To address these problems, we have developed a low-cost, portable image-based cytometer capable of detecting parasitic infections at levels as low as 0.2%. Additionally, our system allows reliable classification of all parasitic life stages at different magnifications. Finally, we demonstrate that the newly developed image-based cytometer can be adopted for antimalarial screening with high accuracy and efficiency and thus can be routinely exploited as a powerful alternative to expensive, technically demanding procedures such as traditional flow cytometry.

## Materials and methods

### Blood collection and parasite culture

Blood used in this project was purchased from Interstate Blood Bank. Before culturing malaria parasites, blood was transferred to EDTA Tubes (VACUETTE^®^ EDTA Tubes, Greiner Bio-One), washed three times in RPMI 1640 (Sigma-Aldrich) by centrifuging at 600g for 10 minutes to remove the buffy coat. RBCs were stored at 50% hematocrit in malaria culture medium (MCM), which is RPMI supplemented with bovine serum (Albumax II, Gibco-Singapore), 2.5 μg/mL gentamycin and hypoxanthine. Standard laboratory strains of *P*. *falciparum*, 3D7, were used in all experiments. Parasites were cultured in human O^+^ erythrocytes at 2.5% haematocrit in MCM under standard conditions [40]. Parasites were synchronized by magnetic selection at late stages (46–48 h) with the help of a SuperMACS magnet (Miltenyi Biotech, Bergisch Gladbach, Germany) and introduced into fresh RBCs, followed by sorbitol synchronization three hours later to allow a tight window of invasion [41].

### Growth inhibition assays and parasitemia determination by flow cytometry and microscopic examination

Parasites at trophozoite stage (24–26 hpi) were treated with a range of concentrations of antimalarial drugs at 2% parasitemia and 2.5% hematocrit. Samples were cultured for 50–52 h until the trophozoites (28–30 hpi) appeared in the next replication cycle and then harvested for further analysis.

Flow cytometry was performed on Accuri C6 flow cytometer (Becton, Dickinson, CA. USA). At least 100,000 RBCs were analysed for each sample to determine parasitemia. To do this, 50 μl aliquots were collected from each sample and fixed with 0.1% glutaraldehyde (Sigma-Aldrich) at 4°C overnight. Fixed cells were collected by centrifuging at 400 g for 3 min, washed in PBS, permeabilized using 0.25% Triton X-100/PBS (Sigma-Aldrich) for 10 min at room temperature and washed again. Further, samples were incubated with 25 μg/ml Hoechst 33342 (Thermo Fisher) for 30 min in dark, and then parasitemia was scored by flow cytometry as reported elsewhere [42]. Data analysis and statistics were performed using GraphPad Prism (GraphPad Software) in accordance with the recommended protocol for nonlinear regression of a log-(inhibitor)-versus-response curve.

Thin smears for microscopic examination were prepared on glass slides and fixed with 100% methanol (Merck). The smears were stained with freshly prepared, filtered 1 in 10 dilution of Giemsa (Sigma-Aldrich) solution in de-ionised water for 10 min. Parasites were observed under an oil immersion lens (100X) using an optical microscope (Leica) for standard experiments.

### Design of in-house image-based cytometer and image acquisition

**Fig 1:**
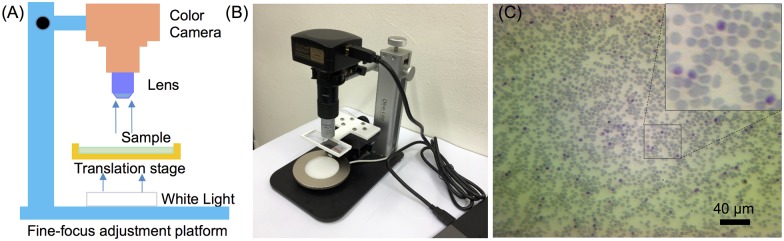
The developed image-based cytometer. (A) Schematic experimental setup of the cytometer. (B) Photograph of the image-based cytometer. (C) An image of the blood smear taken by the cytometer.

A low-cost, portable image-based cytometer was built for image acquisition from Giemsa-stained blood smears. As illustrated in Fig 1A, it consists of a fine-focus (Z axis) adjustment platform (Dino-Lite, Model RK-10 Rack, Singapore), a 14 MP colour camera (ToupTek, P/N:TP114000A, Hangzhou, China), 20X objective or 100X oil immersion objective, two-dimensional translation stage and a white light source with tunable light intensity. Giemsa-stained smears were prepared carefully to obtain uniform cell distribution and placed on the 3D-printed sample holder for imaging. Images were captured with a commercial software (ToupTek, Hangzhou, China) by manually translating the smear slide on the stage. Only fields containing obvious debris were discarded. A 20X objective was used to capture large fields for imaging at high-throughput. However, the use of 20X objective is not sufficient to identify ring stage, in this case, a 100X oil immersion objective was utilized for ring forms imaging and parasitemia analysis.

### Image analysis algorithm

We used image processing and machine learning algorithms to analyse the smear images captured by the developed image-based cytometer. The image analysis algorithm can be divided into three key steps. First, a pre-processing total generalized variation (TGV) denoising method was used to remove the unavoidable noise in the original images. Second, an efficient and robust local adaptive thresholding approach was used to segment the smoothed image with possible intensity inhomogeneity. Finally, a machine learning method was performed to prevent false detections in the case when the image does not contain any parasites. Details of each step will be discussed in the following.

The denoising of the original image was implemented by the TGV method [43], which is a powerful image pre-processing tool that has been extensively used in image processing community [44–46]. The TGV regularisation has the capability of representing image characteristics up to an arbitrary order of differentiation (piecewise constant, piecewise affine, piecewise quadratic etc.). The TGV model is of the following form

$$E\left(u,p\right)=\frac{1}{2\lambda }\overset{}{\underset{\;}{\underset{\Omega }{\int }{\left(u-f\right)}^{2}}}+\underset{\Omega }{\int }\left|\nabla u-p\right|+\alpha \overset{}{\underset{\;}{\underset{\Omega }{\int }\left|\text{ε}\left(p\right)\right|}}$$ (1)

where *E*(*u*,*p*) means the energy functional with respect to two variables *u* and *p*, *u* is the denoised/smoothed image, *p* = (*p*1,*p2*) is a symmetrised gradient vector which is closely related to $\nabla u=\left(\frac{\partial u}{\partial x},\frac{\partial u}{\partial y}\right)$, *f* is the input original image with intrinsic noise, and the operator *ε*(*p*) is the symmetrised derivative which is defined as 0.5(∇*p* + ∇*p*^*T*^). The first energy term on the right hand side of Eq 1 is the data fidelity term which constrains the smoothed image *u* to be similar to the original image *f*. The second term preserves edges of objects in *u* and removes the noise from *f* in the meantime. The third term imposes smoothness on *u* and also eliminates the staircase artefact produced by the second term. The minimum of Eq 1 is taken over all the symmetrised gradient of the deformation field *p* = (*p*1,*p2*) on the image space Ω. The positive *λ* coefficients and α in Eq 1 balance the data fidelity term (the first energy term), the first order derivative (the second energy term) and the second order derivative (the third energy term). The value of α is normally set to 2 and this setting is suitable for most applications [44] and does not need to be tuned. In our experiment, we therefore set α = 2 and only vary *λ* to achieve different smoothness scales.

As TGV takes both first and second derivatives into consideration, it can highlight the edges of objects and smooth image without creating additional artefacts. This property also enables TGV to overcome the problem of intensity inhomogeneity that has been found to widely exist in our captured images. Moreover, since the TGV functional of Eq 1 is convex, it guarantees a global optimal solution as well as allows the use of powerful modern optimization techniques. Finally, TGV is translationally invariant and rotationally invariant, meaning that the denoising results are not affected by the viewpoint of the images taken from different angles. In spite of the outstanding performance of TGV for image denoising, it is difficult to minimise such functional due to its non-linear and non-smooth nature. In this study, we minimise TGV with a fast numerical algorithm based on split Bregman.[47] For a complete implementation on this algorithm, we refer the reader to a previous study [48].

The smoothed image can be then segmented via a simple local adaptive thresholding approach [49], which is given as

$$T=\{\begin{array}{ll} 0, & ls(I,ws)\;-\;I\;>C\\ 1, & otherwise \end{array}$$ (2)

where *T* is the binary image, *I = u* in this case, and *ls*(*I*,*ws*) means that *I* is convolved with a suitable operator, i.e. the mean or median filter. *ws* is the window size of the filter and *C* is a user-defined threshold value. The adaptive thresholding produces binary segmentation with fewer isolated points, giving a better result than a simple high-pass threshold. It is worth mentioning that this approach can segment large sized images (e.g. 2000 × 2000 pixels) with real-time computational speed, making it an ideal segmentation tool for the images used in the experiments. In addition, previous studies have shown that it is robust against inhomogeneity in medical images and can also obtain higher accuracy [50].

To prevent false detections in images that do not contain any parasites, we performed the machine-learning algorithm to automatically classify the parasite and non-parasite images. Specifically, 50 images were first selected manually as a training dataset, and divided into two groups, one containing 25 parasite images and the other group with 25 non-parasite images. The selected representative images were then segmented separately. For each RGB channel of the segmented image, the average intensity of all the pixels that fall in the segmented cells was calculated, thus forming a three-dimensional feature that can be effectively used for training a precise classification model. In this study, we choose Support Vector Machine (SVM) classifier with the linear kernel [51] to train these extracted features. The SVM classifies two different datasets by finding an optimal hyperplane that has the largest margin distance between them. It is more robust and accurate than other machine learning techniques. We therefore used the trained model by the SVM to automatically classify the rest of parasite and non-parasite images.

### Total RBC number and infected RBC number quantification process

**Fig 2:**
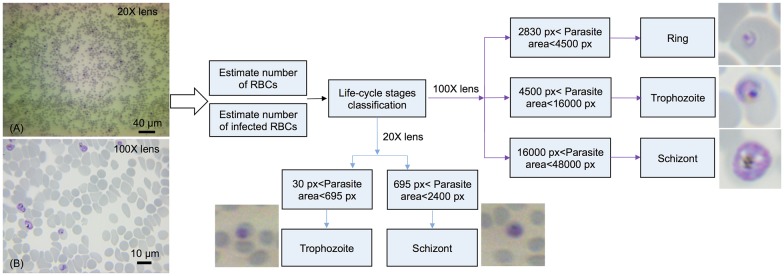
Flowchart of the developed image analysis algorithm. Image A was taken by objective 20X and image B was taken by oil immersion objective 100X.

As shown in Fig 2, the flowchart of the algorithm is composed of three sections: 1) to estimate the total number of all RBCs; 2) to estimate the number of infected RBCs (iRBCs); and 3) to classify distinct developmental stages of the parasite. For images acquired under objective 20X, we used the mean filter with the window size *ws* = 120 and set *C* = 0.03, *λ* = 3 for the segmentation of all RBCs, which produces binary segmentation with isolated RBCs and clustered RBCs. The number of RBCs in the clustered region was estimated by dividing the whole clustered area by the average area of a single RBC. For iRBCs segmentation, a high threshold value *C* = 0.2 was used with *ws* = 100, *λ* = 3. Similarly, under 100X oil immersion objective, *ws* = 300, *C* = 0.01 and *λ* = 5 were used for RBCs segmentation and *ws* = 300, *C* = 0.15 and *λ* = 10 were set for iRBCs segmentation. It should be noted that the *C* value can be affected by the light intensity. After standardizations, we kept a constant, optimum light intensity and fixed exposure time of camera for our further assays. To address the possible risk of counting multiply infected parasites in one single iRBC as multiple iRBCs, we performed the following calculation: if the distance between two segmented parasites is less than 150 pixels, these parasites will be considered as single iRBCs. It might still cause the false classification when two overlapping iRBCs are observed, but this was an extremely rare case due to the careful preparation of thin and uniform smear slides.

### Classification of parasites’ developmental stage

After estimating the number of iRBCs, the algorithm performed the stage classification based on the area occupied by individual parasites within an iRBC. Fig 2 depicts the criteria of parasites’ stage classification under 20X and 100X magnifications. For 100X oil immersion objective, if the area occupied by the parasite is larger than 2830 pixels and less than 4500 pixels, then it was identified as a ring-stage parasite. It was classified as trophozoite stage when the area is greater than 4500 pixels and less than 16000 pixels. If the parasite-occupied area is greater than 16000 pixels and less than 48000 pixels, it was considered as a schizont. For 20X objective, the magnification was insufficient to image the rings, thus we only identify the two late stages under this low magnification. All these threshold pixel numbers for stage classification were verified independently based on conventional cytological examination. The developed system was used to determine parasitemia from seven independent samples. Each sample set included three smears and a minimum number of 30 fields were captured continuously resulting in counting at least 4000 RBCs for each smear. Imaging 30 fields for each smear took about 10 minutes, meaning it took 20 seconds to image each field.

## Results and discussion

### Image analysis results under 20X objective

**Fig 3:**
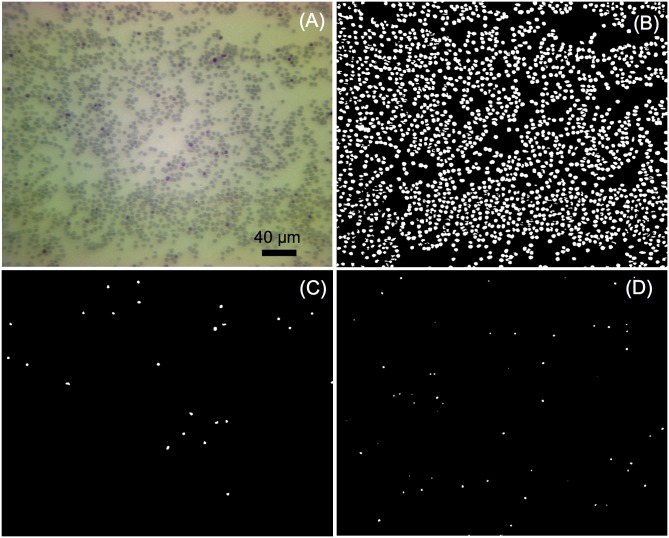
Images automatically analyzed by the developed image-based cytometer. (A) Original image taken by objective 20X. (B) Representation of all the extracted RBCs. (C) Extracted schizont stage iRBCs. (D) Extracted trophozoite stage iRBCs.

The 20X objective was used for high-throughput imaging, and the analyses of images captured by our image-based cytometer were performed automatically using our image analysis algorithm, as shown in Fig 3. Table 1 shows the results from six randomly selected images from the same smear by using the image analysis algorithm and naked eye manual counting. Errors of total RBCs, iRBCs and parasitic classification were less than 5%. These negligible errors of total RBC number were primarily in regions where RBCs were clustered, minimally compromising the counting accuracy. Estimation of the number of iRBCs and life cycle stages were mildly influenced by debris. Nevertheless, error values less than 5% showed the excellent performance and reliability of our image-based cytometer compared to manual counting.

**Table 1:** Comparison of cell counting and developmental stage classification (Objective 20X).

Image No.	1	2	3	4	5	6	Total
**MC (RBCs)**	**1933**	**2381**	**2788**	**2870**	**2821**	**1942**	**14735**
**IBC (RBCs)**	**2000**	**2340**	**2810**	**2753**	**2801**	**1972**	**14676**
**Error (RBCs)**	**3.35%**	**1.72%**	**0.79%**	**4.10%**	**0.71%**	**1.55%**	**4.00%**
**MC (iRBCs)**	**103**	**90**	**127**	**131**	**134**	**78**	**663**
**IBC (iRBCs)**	**106**	**94**	**126**	**131**	**137**	**78**	**672**
**Error (iRBCs)**	**2.91%**	**4.44%**	**0.78%**	**0%**	**2.24%**	**0%**	**1.36%**
**MC (Schizont)**	**50**	**36**	**70**	**63**	**71**	**25**	**315**
**IBC (Schizont)**	**51**	**35**	**72**	**64**	**72**	**25**	**319**
**Error (Schizont)**	**2.00%**	**2.78%**	**2.86%**	**1.59%**	**1.40%**	**0%**	**1.27%**
**MC (Trophozoite)**	**53**	**54**	**57**	**68**	**63**	**53**	**348**
**IBC (Trophozoite)**	**55**	**59**	**54**	**67**	**65**	**53**	**353**
**Error (Trophozoite)**	**3.77%**	**9.26%**	**5.26%**	**1.47%**	**3.17%**	**0%**	**1.44%**

* MC represents the average value of two manual counting performed in the same image which was analysed by image-based cytometer.

* iRBCs—infected Red Blood Cells.

* IBC indicates the result analyzed using the image-based cytometer.

### Image analysis results under 100X oil immersion objective

**Fig 4:**
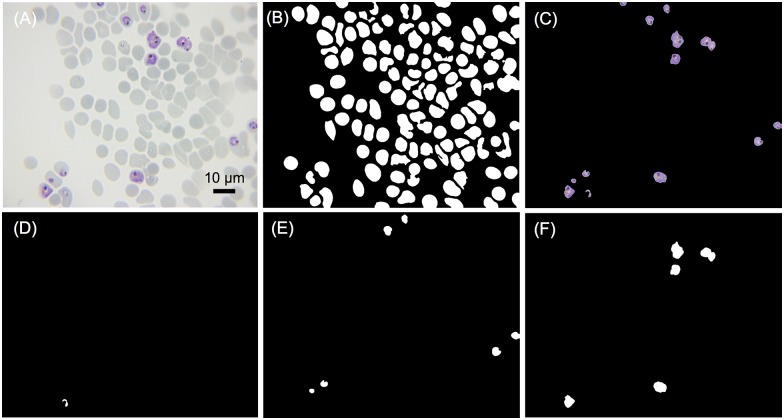
Images automatically analyzed by the developed image-based cytometer. (A) Original image taken by immersion objective 100X. (B) All the extracted RBCs. (C) All the extracted iRBCs. (D) Extracted ring stage. (E) Extracted trophozoite stage. (F) Extracted schizont stage.

Since 20X objective is insufficient to image the ring stages with high accuracy, a 100X oil immersion objective was used for parasitemia detection and classification. As illustrated in Fig 4, image-based cytometer equipped with 100X allowed us to reliably classify all the three parasitic stages. Table 2 shows the inspection results of RBCs, iRBCs segmentation and stage-specific classification. The ability of our proposed cytometer to classify and document different developmental stages may lead to potential pharmacological applications in identifying the potential blockers of egress, invasion and parasitic development inhibitory molecules [38]. In a single image, errors of a specific parasite stage may be large (due to limited number of parasites in the field), but the total error of randomly selected 6 images was well below 5%, which shows the robustness of our system. Giemsa-stained images taken at 20X and 100X objective lens in the aforementioned studies are shown in S1 and S2 Figs of supplementary information, respectively.

**Table 2:** Comparison of cell counting and developmental stage classification (Immersion objective 100X).

Image No.	1	2	3	4	5	6	Total
**MC (RBCs)**	189	132	170	182	122	160	955
**IBC (RBCs)**	183	133	162	184	123	165	950
**Error (RBCs)**	3.17%	0.76%	4.71%	1.10%	0.82%	3.13%	0.52%
**MC (iRBCs)**	10	13	9	8	9	7	56
**IBC (iRBCs)**	10	13	8	8	9	7	55
**Error (iRBCs)**	0%	0%	11.10%	0%	0%	0%	1.79%
**MC (Schizont)**	6	5	5	4	5	4	29
**IBC (Schizont)**	6	5	4	4	6	4	29
**Error (Schizont)**	0%	0%	20%	0%	20%	0%	0%
**MC (Trophozoite)**	4	7	4	4	3	1	23
**IBC (Trophozoite)**	4	7	4	3	2	2	22
**Error (Trophozoite)**	0%	0%	0%	25%	33.30%	100%	4.35%
**MC (Ring)**	0	1	0	0	1	2	4
**IBC (Ring)**	0	1	0	1	1	1	4
**Error (Ring)**	0%	0%	0%	100%	0%	50%	0%

* MC represents the average value of two manual counting performed in the same image which was analysed by image-based cytometer.

* iRBCs—infected Red Blood Cells.

* IBC indicates the result analyzed using the image-based cytometer.

### Estimation of false positives

**Fig 5:**
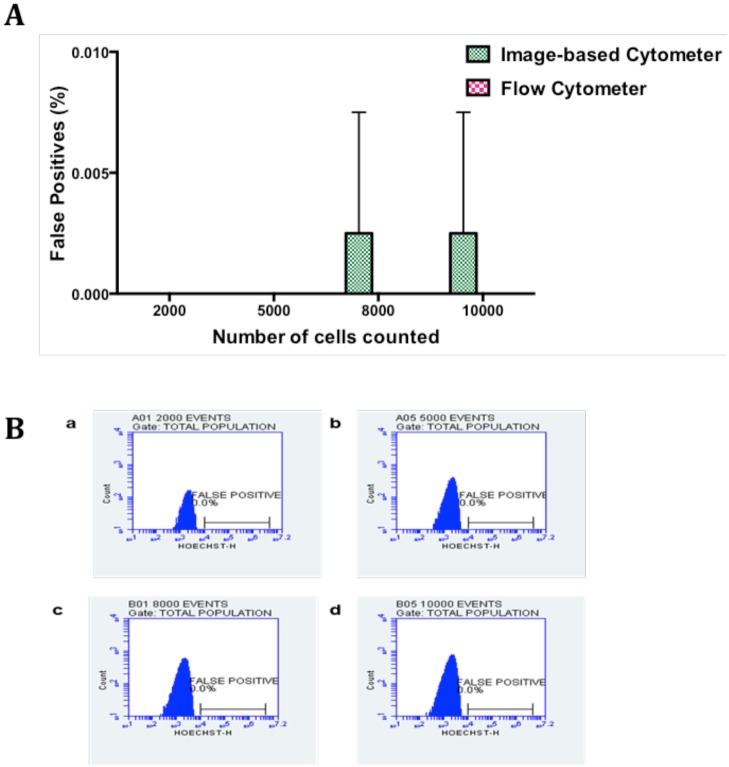
Comparison of false positives in hRBCs using image-based cytometer and flow cytometer. (A) Briefly, freshly drawn RBCs were counted and scored using image-based cytometer and flow cytometer, respectively. There were no false parasites obtained until 5000 RBCs were scored using image-based cytometer. Our developed cytometer exhibited 0.0025% false positive values while calculating more than 5000 cells. The figure represents the average of two independent experiments performed in duplicates and the error bars represent the standard deviation of the average values. (B) The figure represents the histogram plots for false positives estimated using flow cytometer while counting 2000 (a), 5000 (b), 8000 (c) and 10000 (d) events.

**Fig 6:**
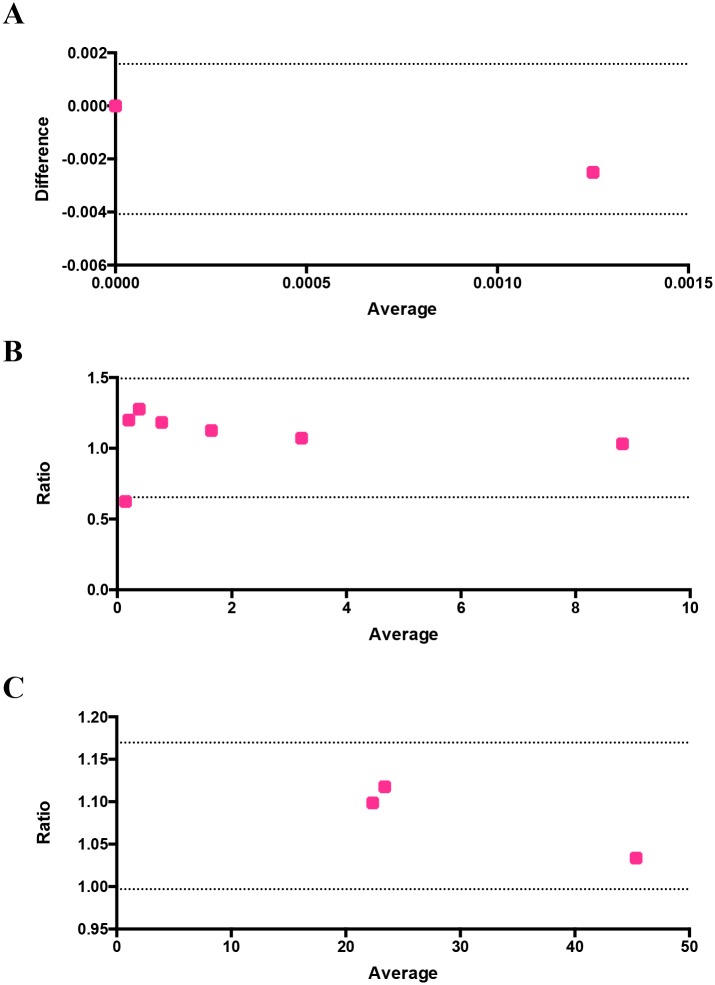
Evaluation of the performance and reliability of the image-based cytometer using Bland-Altman method of comparison. The differences between the false positives of flow cytometer and image-based cytometer versus their average is plotted in (A). Ratio of parasitemia detected through flow cytometer and image-based cytometer and ratio of IC_50_ values identified through flow cytometer and image-based cytometer are shown in (B) and (C) respectively. All values are within the 95% limits of agreement, which are shown as two dotted lines in the figures.

There are several factors that could account for the sensitivity of the microscopic examination of thin blood smears such as quality of the smears (uniform dispersion of single cells vs clump of cells), duration and dilution of Giemsa staining, and debris and dirt arising from Giemsa solution [52]. Previous reports have shown a huge variation in parasitemia levels between 6.3% and 45.8% based on 48 microscopic slides circulated between four laboratories in Mpumalanga province of South Africa [53]. We therefore set up trial experiments to standardize the parameters such as the dilution and duration of Giemsa staining, uniform distribution of RBCs to reduce and prevent the false positive values. From our trial experiments, we noticed that uniform distribution of RBCs stained with 1 in 10 dilution of Giemsa in distilled water for 10 minutes and washed with distilled water showed the best results. To determine the false positive value of image-based cytometer, healthy RBCs (hRBCs) were diluted in MCM and same number of RBCs (ranging from 2000 to 10000) was scored using both flow cytometer and our image-based cytometer. As expected, there was an absolute zero detection of false positive value in the flow cytometer and the corresponding flow plots are shown in Fig 5B and the image-based cytometer showed 0.0025% false positive detection for 8000 and 10000 cells. As shown in Fig 5A, there were no false positives detected using image-based cytometer when calculating cells less than 5000. In order to calculate the false positives, we performed two independent experiments in duplicates. In one of the replicates of the first independent experiment, we noticed 1 false positive while counting 8000 and 10000 cells, which accounts for 0.0125% and 0.01% false positives. We rounded off our results to decimal places, getting 0.01% false positive value and 0.0025% average false positive value in total for two independent experiments in duplicates and the differences between the values of image-based cytometer and flow cytometer were found to be nonsignificant using two tailed Wilcoxon T test. The result obtained from this experiment was evaluated using Bland-Altman method of comparison and it is shown in Fig 6A. It should be noted that the values are within the 95% limits of agreement (lower 95% limit of agreement = -0.004079 and upper 95% limit of agreement = 0.001579).

### Pharmacological testing using the developed image-based cytometer

Reliable, easy and affordable methods of inexpensive and high throughput screening techniques are needed for quantitative evaluation of new classes of antimalarials. Quantification of radioactive substance uptake such as [^3^H]hypoxanthine [54], [^3^H]isoleucine [55] and [^3^H]ethanolamine [56] by malaria parasites in the presence of the drugs have been measured to assess the drug efficacy. Several tools including flow cytometry and fluorescence microscopy are commonly employed for antimalarial tests in research laboratories. Here, we investigated the application of our image-based cytometer in pharmacological assays using three well-established antimalarials: chloroquine, artemisinin and cycloheximide in comparison to a more laborious flow cytometry method. These three antimalarials were selected since they act through distinct mechanisms and halt the parasitic growth and development under different stages [57].

**Fig 7:**
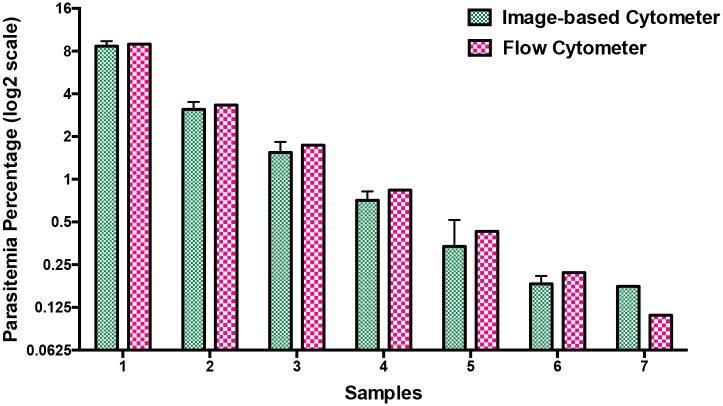
Comparison of parasitemia values using image-based cytometer and flow cytometer. Mixed stage malaria parasites were diluted in hRBCs to obtain parasitemia ranging between 0.2% and 9% and scored using image-based cytometer. The results were comparable with flow cytometer values with no significant difference. The figure represents the average of three independent measurements performed and the error bars represent the standard deviation of the average values.

Prior to proceeding with drug testing, we evaluated the potency of our image-based cytometer over a range of parasitemia and the results were compared against flow cytometry. Mixed stage parasites were diluted to obtain parasitemia ranging between 0.2% and 9%. Giemsa-stained smears were taken for assessing the parasitemia (in a blind manner to not reveal sample identity) using image-based cytometer in parallel to flow cytometry-based assay. As shown in Fig 7, parasitemia scored using our image-based cytometer were totally comparable to the values obtained from flow cytometric experiments as confirmed through two-tailed paired T test. Corresponding flow cytometer plots are shown in S3 Fig of the supplementary information. The results obtained through this parasitemia determination experiment is evaluated using Bland-Altman method of comparison and it is shown in Fig 6B. It should be noted that the values are within the 95% limits of agreement (lower 95% limit of agreement = 0.6539 and upper 95% limit of agreement = 1.493).

**Fig 8:**
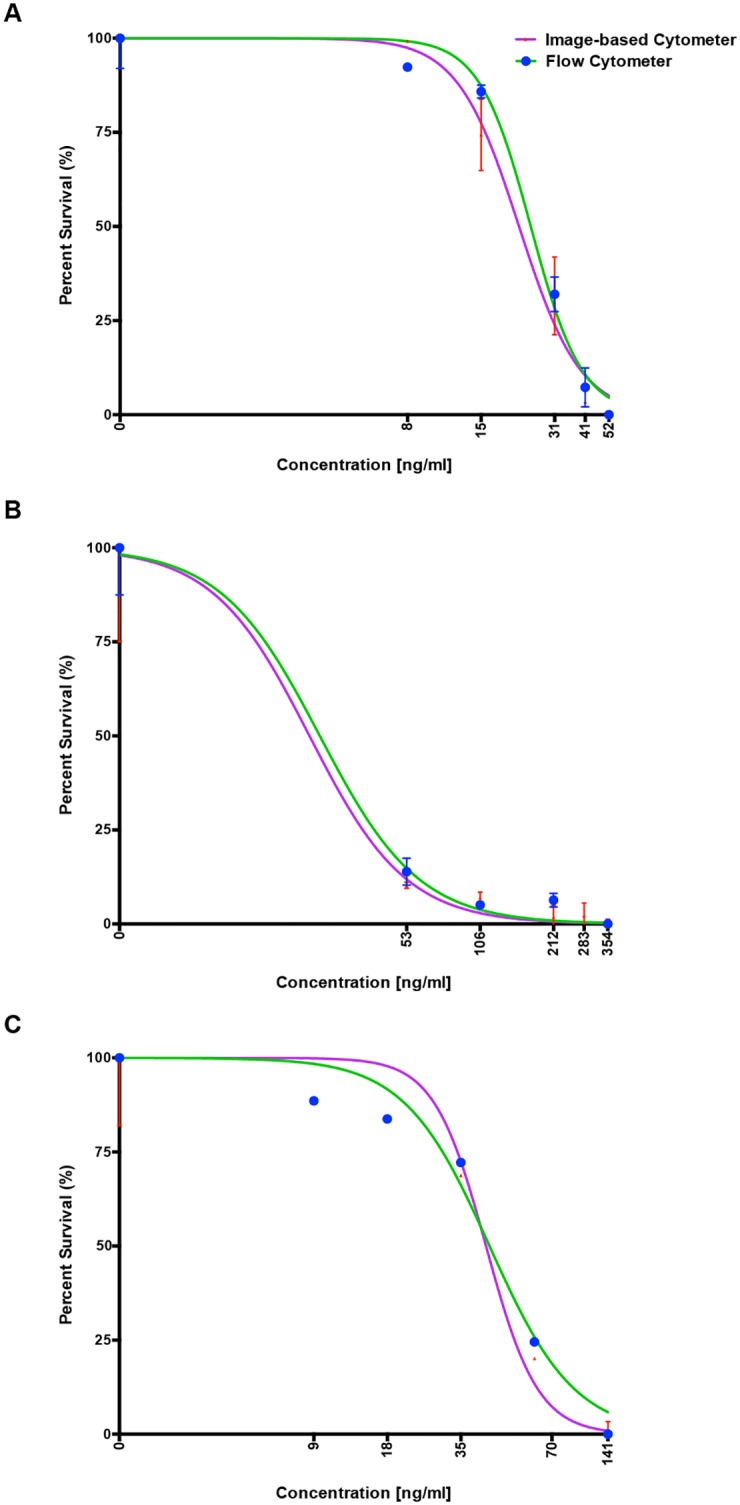
Comparison of dose response curves for the three antimalarial drugs generated using image-based cytometer and flow cytometer. Well-known anti-malarials; (A) Chloroquine, (B) Artemisinin and (C) Cycloheximide were screened against the laboratory strain, 3D7. Standard growth inhibition assay was performed and IC_50_ values determined using our image-based cytometer and flow cytometry in parallel [Table 3], to obtain comparable results. The error bars represent standard errors.

Next, dose-response curves for chloroquine, artemisinin and cycloheximide estimated through image-based cytometer and standard flow cytometer are shown in Fig 8. Despite minor differences, IC_50_ values defined as half maximal inhibitory concentration calculated through these methods were similar (and mostly in agreement with reported values elsewhere [57–59]) as listed in Table 3. These results demonstrate the feasibility of our proposed system for inexpensive, highly sensitive and reliable, high throughput screening of antimalarials. The IC_50_ values obtained by the image-based cytometer was evaluated using Bland-Altman method of comparison and it is shown in Fig 6C. It should be noted that the values are within the 95% limits of agreement (lower 95% limit of agreement = 0.9970 and upper 95% limit of agreement = 1.170).

**Table 3:** Comparison of IC_50_ determined by two different methods.

Antimalarial drugs	IC_50_(Flow cytometer)	IC_50_(Image-based cytometer)
**Chloroquine (ng/ml)**	24.7	22.1
**Artemisinin (ng/ml)**	23.4	21.3
**Cycloheximide (ng/ml)**	46.1	44.6

## Conclusions

In this study, we have developed an image-based cytometer for scoring and classifying the stages of malaria parasites. This portable system, which is a combination of commercial parts and in-house 3D-printed parts, has a compact dimension of 22 cm (L) x 15 cm (W) x 23 cm (H) and weights less than 2000 grams and can be used in routine laboratory drug screening and potential on field-diagnosis. By comparing the results obtained by our developed method to manual counting and flow cytometry, we have validated its robustness and accuracy to differentially estimate all parasitic stages. For the parasitemia test and IC_50_ determination assays, results were comparable with flow cytometry method. Several advantages of our portable image-based cytometer are summarized as follows: 1) it provides comparable results to flow cytometry and allows stage-specific scoring of parasitemia; 2) it is a non-fluorescent method with simple preparation and operation, exhibiting high computational speed less than 10 seconds per image (Dimensions: 4096 x 3286) using commonly used laptops; 3) this image-based cytometer is low-cost (less than 600 USD) and compact, therefore making it a reliable and affordable tool to (i) detect and score level of infection and (ii) facilitate screening of antimalarials from natural or synthetic products in resource-poor settings.

## Supporting information

S1 Fig Six randomly selected images from the same smear taken and analysed using the developed image-based cytometer under 20X objective lens. (Quantitative analyses are shown in the Table 1).
(TIFF)

S2 Fig Six randomly selected images from the same smear taken and analysed using the developed image-based cytometer under 100X oil immersion objective lens. (Quantitative analyses are shown in the Table 2).
(TIFF)

S3 Fig Comparison of true positives (parasitemia) measured using image-based cytometer and flow cytometer. The figure represents the histogram plots of parasite values calculated using flow cytometer. Figures (a) to (g) represents the different samples from 1 to 7 showed in Fig 7.
(TIFF)
