# Supplementary figures and images for: A portable image-based cytometer for rapid malaria detection and quantification
Source: PLoS One. 2017 Jun 8;12(6):e0179161. doi: 10.1371/journal.pone.0179161 (PMC5464641; doi:10.1371/journal.pone.0179161)

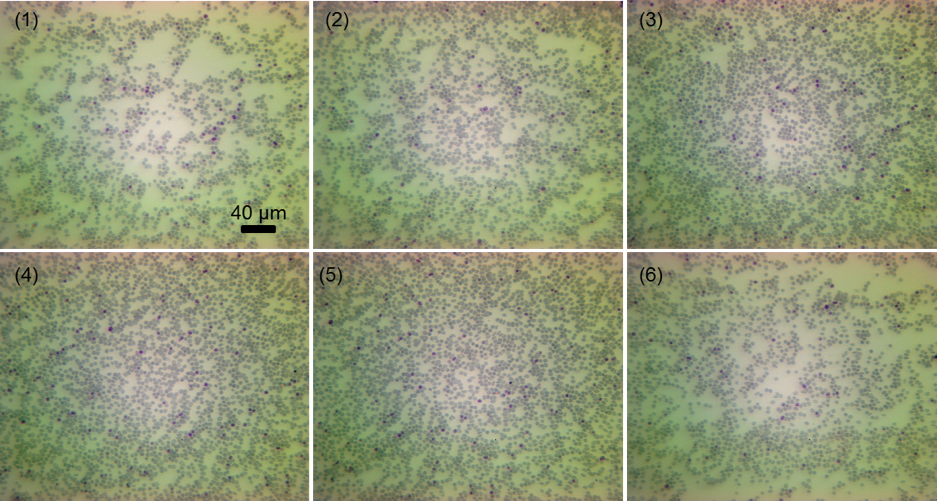

Supplement: S1 Fig — (Quantitative analyses are shown in the Table 1). (TIFF) [file pone.0179161.s001.tiff]

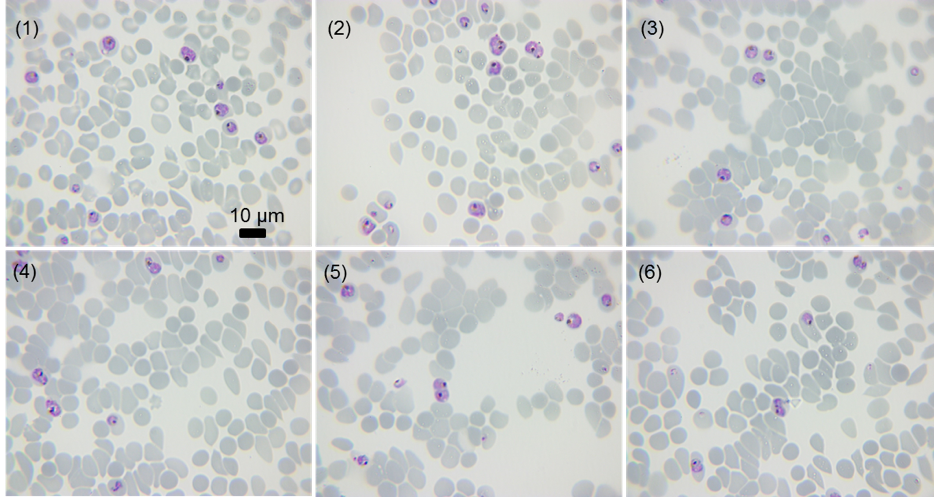

Supplement: S2 Fig — (Quantitative analyses are shown in the Table 2). (TIFF) [file pone.0179161.s002.tiff]

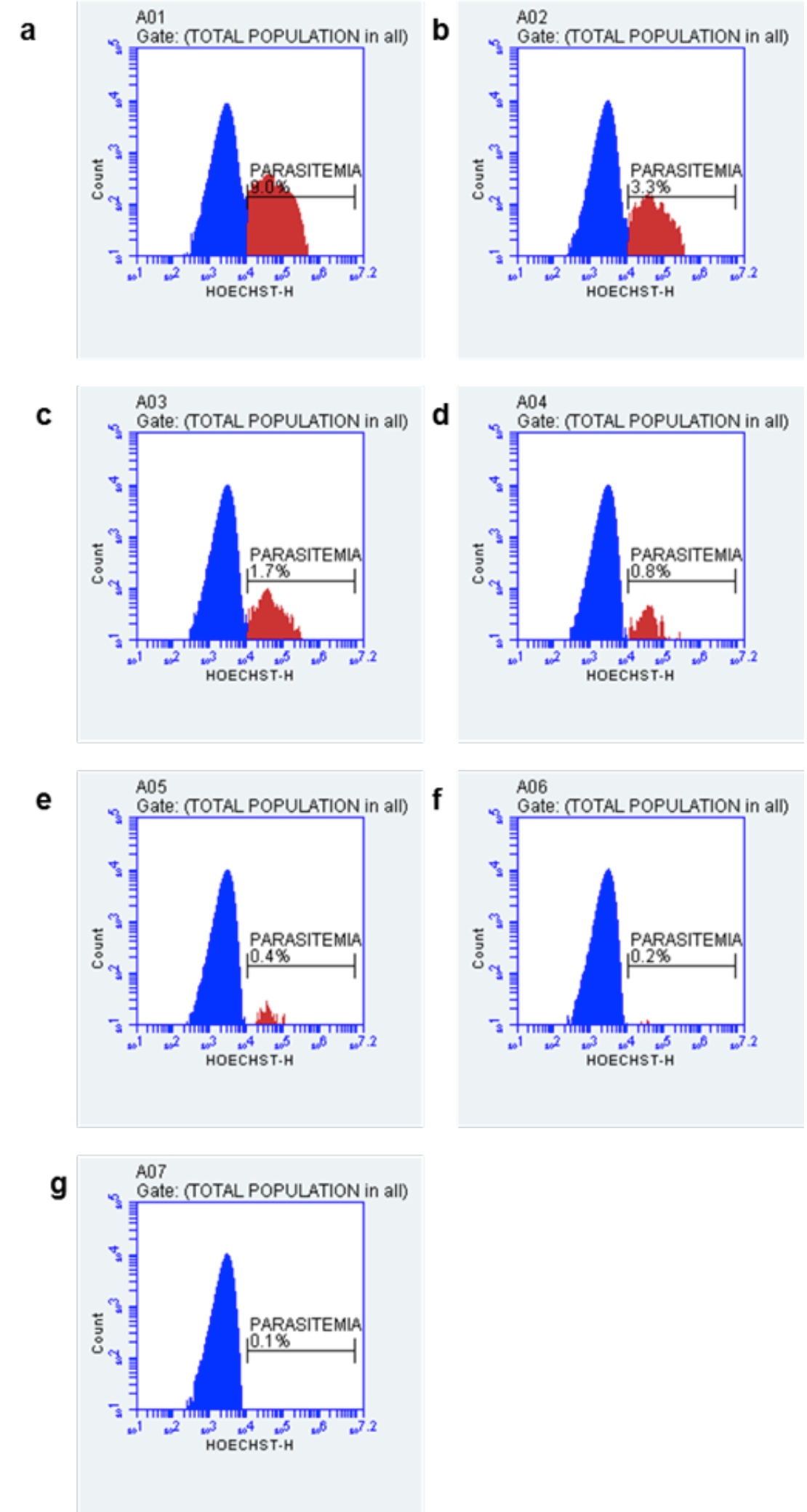

Supplement: S3 Fig — The figure represents the histogram plots of parasite values calculated using flow cytometer. Figures (a) to (g) represents the different samples from 1 to 7 showed in Fig 7. (TIFF) [file pone.0179161.s003.tiff]
